# Analysis of Dehydration and Strength in Elite Badminton Players

**DOI:** 10.1371/journal.pone.0037821

**Published:** 2012-05-29

**Authors:** Javier Abián-Vicén, Juan Del Coso, Cristina González-Millán, Juan José Salinero, Pablo Abián

**Affiliations:** Exercise Physiology Laboratory, Camilo José Cela University, Madrid, Spain; University of Granada, Spain

## Abstract

**Background:**

The negative effects of dehydration on aerobic activities are well established. However, it is unknown how dehydration affects intermittent sports performance. The purpose of this study was to identify the level of dehydration in elite badminton players and its relation to muscle strength and power production.

**Methodology:**

Seventy matches from the National Spanish badminton championship were analyzed (46 men’s singles and 24 women’s singles). Before and after each match, jump height and power production were determined during a countermovement jump on a force platform. Participants’ body weight and a urine sample were also obtained before and after each match. The amount of liquid that the players drank during the match was also calculated by weighing their individual drinking bottles.

**Results and Discussion:**

Sweat rate during the game was 1.14±0.46 l/h in men and 1.02±0.64 l/h in women. The players rehydrated at a rate of 1.10±0.55 l/h and 1.01±0.44 l/h in the male and female groups respectively. Thus, the dehydration attained during the game was only 0.37±0.50% in men and 0.32±0.83% in women. No differences were found in any of the parameters analyzed during the vertical jump (men: from 31.82±5.29 to 32.90±4.49 W/kg; p>0.05, women: from 26.36±4.73 to 27.25±4.44 W/kg; p>0.05). Post-exercise urine samples revealed proteinuria (60.9% of cases in men and 66.7% in women), leukocyturia (men = 43.5% and women = 50.0%) and erythrocyturia (men = 50.0% and women = 21.7%).

**Conclusions:**

Despite a moderate sweat rate, badminton players adequately hydrated during a game and thus the dehydration attained was low. The badminton match did not cause muscle fatigue but it significantly increased the prevalence of proteinuria, leukocyturia and erythrocyturia.

## Introduction

The negative effects of dehydration on endurance sports have been widely demonstrated [1]. A loss of over 2% of body mass has a negative effect on physical performance in a variety of aerobic sports activities [1]–[3]. However, the effects of dehydration on short-term high-intensity activities are less clear. While some studies indicate that dehydration does not affect short-term muscle performance [4], [5] other investigations have found a detrimental effect of dehydration on maximal force production [6] and high-intensity cycling performance [7], especially when dehydration is accompanied by hyperthermia. In a recent review, Judelson et al. [8] indicate that dehydration has a greater influence the longer the exercise lasts. However, these authors also indicate that a reduction of 3–4% of body mass can reduce muscle strength by 2%, muscle power by 3% and muscle endurance during efforts of between 30 and 120 seconds by 10%. Frequently, the dehydration level attained during a sport activity depends on sweat rate, duration and fluid intake.

The sweat rate recorded in a sport is conditioned by a series of factors like exercise intensity, training level, heat-acclimatization, environmental conditions, clothing or even the facility where the sport is played (indoor or outdoor sports). Maughan et al. [9] measured the sweat rate at different environmental temperatures in elite soccer players. They found that at 5°C the players had a sweat rate of 1.1 l/h, at 25°C of 1.2 l/h and at 32°C of 1.5 l/h. Tippet et al. [10] found a sweat rate in tennis players of 2.0±0.5 l/h during a tournament with an environmental temperature of 30.3±2.3°C. However, in indoor sports environmental conditions can be controlled, like water vapor pressure (humidity) and environmental temperature (dry temperature), so the sweat rate will be mainly influenced by the intensity of effort [11]. Another factor affecting sweat rate is the continuity of the sports (continuous *vs* intermittent sports). In some intermittent sports like soccer [12] and basketball [13] similar sweat rates have been found (from 0.6 to 1.6 l/h) to those attained in cyclic sports like cycling or running [14], [15]. This is because in spite of there being pauses, the moments of activity are of a higher intensity.

Badminton is a non contact racket sport which requires jumping, changing direction, rapid arm movements and a wide range of body postures [16]. Badminton can be considered an intermittent individual sport, characterized by combining moments of high intensity interspersed with short periods of low intensity or rest. In badminton, as in the rest of the racket sports, the players have many opportunities to rehydrate during play due to the intervals between points and sets [17], but does the player start the match adequately hydrated? By measuring the specific gravity of the urine (U_sg_) several studies have found that in sports like athletics, volleyball, basketball or soccer, a large percentage of players arrive at the match with high levels of dehydration before the start of play [18]–[20]. Urine analysis is a method which has been used in several studies to determine changes in fluid balance [20], [21] and to observe the response of the organism to physical exercise [22]. Different studies have found that strenuous exercise produces abnormalities in the players’ urine like modifications in the pH value, hematuria or proteinuria [22]–[25]. However, it should be mentioned that these abnormalities present in the players’ urine after exercise are often asymptomatic and cause no subsequent renal problems [26].

We have found no previous study that analyses the influence of a competitive match on muscle performance in badminton players. Current measuring systems permit assessment of dynamic muscle force and power in real competitive situations with high validity and reliability. But in spite of this there are no published reference values for muscle strength and power, or their relation with the dehydration attained during a badminton match. Studies have been carried out on these kinetic parameters in other racket sports, mainly tennis, and have found hand grip strength values which vary between 400 and 500 N in men and between 300 and 400 N in women, depending on the characteristics of the players studied [27], [28].

The purpose of this study was to identify how a badminton match affects elite badminton players with regard to dehydration, liquid replacement, muscle strength and power using a jump and hand grip tests. We also investigated sex differences in these parameters. The analysis of these parameters may be helpful for players, trainers and physiologists to recommend strategies for liquid replacement during a badminton game.

## Materials and Methods

### Sample

Seventy players, 46 who participated in the men’s singles matches (MS) and 24 who participated in the women’s singles matches (WS) of the national Spanish championship voluntarily participated in the study. At the time of the championship, all participants were among the top 60 players in the National Ranking in the modality of men’s singles and women’s singles respectively. The men’s group had a mean ± SD age of 22.7±4.2 yrs, body mass of 74.54±8.02 kg, height of 178±8 cm, body fat percentage of 8.4±1.4%, body muscle percentage of 50.2±1.3% and training of 13.3±9.2 hours per week. The women’s group had an age of 23.0±5.7 yrs, body mass of 59.45±3.37 kg, height of 165±2 cm, body fat percentage of 16.9±2.4%, body muscle percentage of 46.5±2.0% and training of 16.5±11.6 hours per week. All participants were informed about the nature and the purpose of the study, as well as the measurements which were going to be taken. After that, participants signed a consent form to allow the researchers to take the measurements and use their data for scientific purposes. The study was approved by the local Research Ethics Committee, in accordance with the latest version of the Declaration of Helsinki [29].

### Protocols

Three weeks before the Spanish Badminton Championship all the players who were going to take part in the men’s singles and women’s singles were informed about the purpose of the research and were encouraged to participate, so that when they arrived at the sports centre on the Friday (the day the competition started) they were asked to go to the Stand which had been prepared at the side of the multisport court. On arrival, the participants gave a urine sample and had their descriptive variables measured. Height was measured with a SECA personal height meter (SECA Ltd, Germany) with an accuracy of ±0.05 cm; body mass with a ±0.05 kg scales (Radwag, Poland) and a record was made of the number of hours they trained per week. The participants were familiarized with the measuring instruments corresponding to the maximal intensity tests (countermovement jump and hand grip strength) and they signed the informed consent document.

It was agreed with the competition’s main referee that the players would be told 10 minutes before being called to the court so that once they had performed their warm up they could pass by the Stand and have the pre-game measurements taken without interfering with the normal course of the competition. Immediately before each match (after having performed their routine warm up) and as soon as the game was over, the players were weighed wearing the clothes they had worn during the game (shorts and short sleeved shirt) and then performed two maximum countermovement jumps (CMJ) and a hand grip strength test with each hand (right and left). The drinks that the players took to the court were also weighed before and after the match with a portable scale with ±1 g sensitivity (Tanita KD400, Japan) to calculate liquid replacement. The mean dry temperature measured during the day of the tests was 24±3°C (range: 22–27°C) while relative humidity was 50±6% (range: 42–58%).

In the CMJ test the subject jumped on a Quattro Jump force platform (Kistler, Switzerland) with their hands on the waist at all times. The angle of knee flexion during the CMJ was freely chosen by the subject. The highest jump recorded out of two valid attempts with a 1 min rest between them was chosen for the analysis. A sampling frequency of 500 Hz was used for the recording. In the hand grip strength test, the subject had to grip a manual dynamometer (Takei Scientific Instruments Co. Japan) as hard as possible. Two attempts were made with the elbow extended, the arm parallel to the body and the wrist in neutral position according to the indications of several authors [30], [31]. There was 1 minute of rest between attempts and the highest value was chosen for the analyses.

Urine samples were also collected before and after each match. Each player was given a sterile container and gave a representative mid-stream urine sample. The samples were collected and analyzed fresh within an hour of collection. The measurement was made by introducing a reactive strip (Combur Test®, Roche, Spain) into a small portion of the urine sample so that the components of the sample could react with the reactive agents on the strip. Later, the strip was introduced into an automatic reflection photometer which measured the parameters after an incubation of 1 minute (Urisys 1100, Roche, Spain) [32]. The strips had reactive agents for 10 variables so that after incubation a detector measured the light reflected on each reactive area. The variables measured in the urine were: specific gravity (U_sg_), pH, protein, glucose and ketone body concentration, and the presence of erythrocytes and leukocytes.

### Variables

The following descriptive variables were recorded: age (years), body mass (kg), height (cm), and hours of training per week. The dependent variables analyzed in the matches were: body mass (kg), percentage dehydration calculated from the difference in body mass before and after the match and taking as the reference the mass before the match (%), the quantity of liquid that the players drank during the match (l/h), the duration of the match (min), the height of the jumps calculated from the flight time (cm), mean power during the push-off phase of the CMJ normalized for the mass of the player (W/kg) and hand grip strength of the dominant and non dominant hand (N). The dependent variables analyzed in the urine samples were: specific gravity (U_sg_), pH, leukocytes (leukocytes/µl), nitrites (positive-negative), proteins (mg/dl), glucose (mg/dl), ketone bodies (mg/dl), urobilinogen (mg/dl), bilirubin (mg/dl), and erythrocytes (erythrocytes/µl).

The independent variables were established as the type of match (MS  =  men’s singles; WS  =  women’s singles) and the moment of testing (pre  =  before the match; post  =  after the match).

### Statistics

The following software programs were used: *Microsoft Excel spreadsheet* (Microsoft, Spain) to store the results and the *SPSS v. 17.0* program (SPSS Inc., USA) to perform the statistical calculations using descriptive, inferential and normality statistical tests and to calculate means, standard deviations and ranges. A two way ANOVA (2×2) for repeated measures was used as the inferential test to analyze pre-post and gender differences, the first factor being the moment of testing (pre-post) and the second the match modality (MS-WS). When there were significant differences the Bonferroni post-hoc test was applied. In tests where measurements were not taken before and after the match as in the case of hydration, percentage of dehydration and match duration, Student’s t test for independent samples was used to establish the differences between the different match modalities. The McNemar test for related proportions was used to analyze the differences before and after each match in the dichotomic variables which were revealed in some urine parameters. The criterion for statistical significance was set at p<0.05.

## Results

A sweat rate of 1.02±0.61 l/h was recorded in the women and 1.14±0.46 l/h in the men during the badminton game. The rate of fluid intake in the WS was 1.01±0.44 l/h compared with 1.10±0.55 l/h in the MS matches. The MS matches lasted on average 7.66 minutes longer than the WS matches (WS = 35.08±11.72 min, MS = 42.74±11.30; p<0.05). There was a significant loss of mass from the beginning of the match to the end in both the WS (Pre = 60.7±4.1 kg, Post = 60.5±4.1; p<0.05) and the MS (Pre = 74.4±7.2 kg, Post = 74.1±7.2; p<0.05) which represented a dehydration of 0.32±0.83% in the former and 0.37±0.50% in the latter.

**Figure 1:**
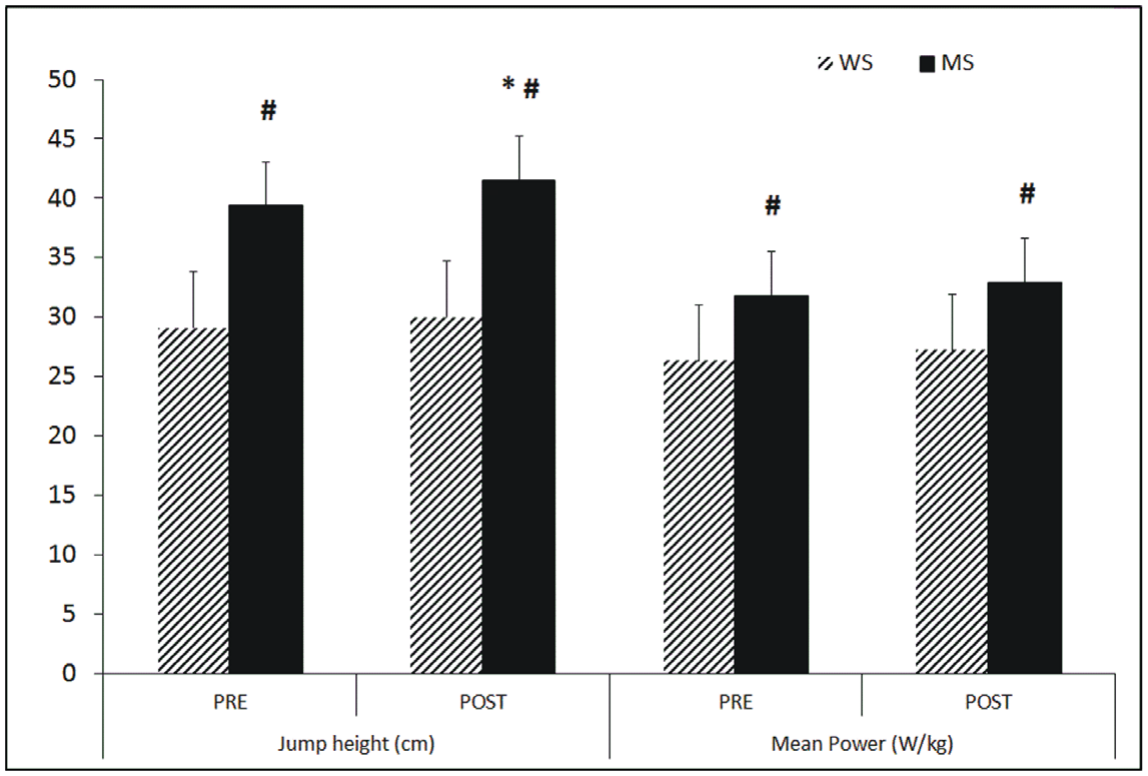
Countermovement jump variables. WS  =  Women’s singles, MS  =  Men’s singles, #  =  significant differences p<0.05 obtained comparing men’s singles with women’s singles.

**Figure 2:**
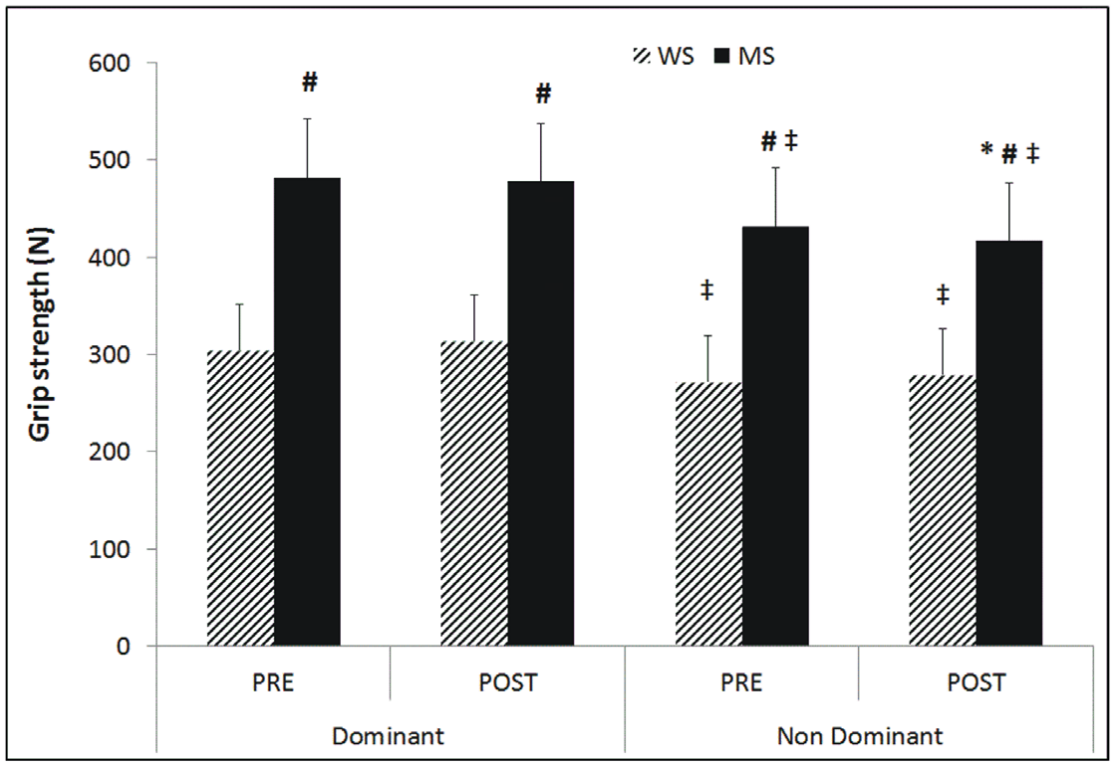
Hand grip strength test variables. WS  =  Women’s singles, MS  =  Men’s singles, *  =  significant differences p<0.05 obtained comparing pre and post match measurements, #  =  significant differences p<0.05 obtained comparing men’s singles with women’s singles, ‡  =  significant differences p<0.05 obtained comparing hand grip strength of the right hand with the left hand.

The results obtained in the variables analyzed for the CMJ and hand grip strength tests are shown in Figures 1 and 2. There was an increase of 4.5±7.3% (p<0.05) in the height of the jumps after the MS matches. Higher values were observed in the men’s group than in the women’s group in the mean power of the push-off phase and in hand grip strength both in the right and the left hand. Considerable differences were found in the subjects’ hand grip strength between the dominant and non dominant side (Figure 2).

There was a significant decrease in the urinary pH values in the men’s group after the match (pre = 7.20±1.08, post  = 6.28±1.05; p<0.05). There was also a decrease in the women’s group although it was not significant (pre = 7.20±1.21, post = 6.25±0.87; p = 0.059). An increase was observed in the nitrite and protein concentration after the match in both the men’s and women’s groups (Table 1). Before the match 0.4% of the men were positive for nitrites compared to 52.2% after the match. The tendency in the women’s group was similar, no subjects showed positive in this test before the start of the competition, but 58.3% showed positive after it. Before the competition only 10.0% of the women and 8.6% of the men had a urine protein concentration ≥25 mg/dl; however, after the match these values increased to 66.7% in the women and 60.9% in the men, with 34.8% of the men revealing values of ≥150 mg/dl. There was also an increase in glucose concentration in the men at the end of the match, 17.4% of whom had values ≥50 mg/dl. The percentage of subjects who had a value of erythrocytes of over 10 erythrocytes/µl also increased. Significant differences were not found in the remaining variables (Table 1).

**Table 1:** Percentage of cases showing values presented for each of the urinary parameters analyzed before (pre) and after (post) the match.

		WS		MS	
		PRE (%)	POST (%)	PRE (%)	POST (%)
	25	20.0	33.3	4.3	39.1
**Leukocytes**(leukocytes/ml)	≥100	20.0	16.7	0.0	4.3
	**% accumulated**	**40.0**	**50.0**	**4.3**	43.5*
**Nitrites**	**Positive**	**0.0**	58.3*	**0.4**	52.2*
	25	10.0	16.7	8.6	17.4
**Proteins**(mg/dl)	75	0.0	33.3	0.0	8.7
	≥150	0.0	16.7	0.0	34.8
	**% accumulated**	**10.0**	66.7*	**8.6**	60.9*
**Glucose**(mg/dl)	**≥50**	**0.0**	**0.0**	**0.0**	17.4*
**Ketone bodies** (mg/dl)	**≥5**	**0.0**	**0.0**	**8.6**	**17.4**
**UBG** (mg/dl)	**≥1**	**10.0**	**25.0**	**30.4**	**39.1**
**Bilirubin** (mg/dl)	**≥1**	**10.0**	**33.3**	**30.4**	**39.1**
**Erythrocytes** (Erythrocytes/µl)	**≥10**	**20.0**	50.0*	**4.3**	21.7*

(WS  =  Women’s singles, MS  =  Men’s singles, *  =  significant differences p<0.05 obtained comparing pre and post match measurements).

## Discussion

### Hydration and Fluid Replacement

The sweat rate of players in an elite competitive badminton match was 1.02±0.61 l/h in the women and 1.14±0.46 l/h in the men. The matches used for the present study were played at a mean neutral environmental temperature of 24±3°C. Badminton is an indoor sport and contrary to what happens in outdoor sports, environmental conditions can be controlled (mainly humidity and dry temperature). In the case of indoor sports, sweat rates are principally influenced by intensity of effort and the pauses in the game [11]. The sweat rate recorded in the present study was similar to that found by Maughan et al. [9] when they analyzed elite soccer players at a temperature of 25°C, similar to the temperature recorded in our study. However the sweat rate was lower than that recorded by different authors whose studies were performed at higher temperatures. Maughan et al. [9] recorded 1.5 l/h in soccer players at an environmental temperature of 32°C and Tippet et al. (2011) [10] measured a sweat rate of 2.0±0.5 l/h during a tennis tournament played at an environmental temperature of 30.3±2.3°C, 6°C higher than the temperature recorded in the present study.

The sweat rate recorded in the badminton matches was slightly lower than that found by Hamouti et al. [18]. These authors analyzed sweat rate in collective acyclic indoor sports like soccer, basketball, volleyball and handball, in similar conditions to ours (21±2°C) with volleyball and handball revealing the lowest sweat rates (1.2±0.3 l/h) while indoor soccer showed the highest sweat rate with a mean of 1.8±0.7 l/h. Badminton, with a small court and play characteristics which involve a multitude of jumps and changes of direction over short distances could be most likened to volleyball, in which similar sweat rates were found.

The value for dehydration recorded in the WS was 0.32±0.83% and 0.37±0.50% in MS. Twelve percent of the players had a dehydration level higher than 1%, only one of these had a value higher than 2% while 66% of the players revealed values between 0–1%; and 22% presented hyperhydration, which in no case was higher than 1%. To summarize, the hydration habits shown by the badminton players were adequate to prevent the 2% dehydration which can cause a decrease in sports performance [1]–[3]. The rest intervals which occur during a badminton match favor the adequate hydration of the players, as each player has a 60 second rest when the first player reaches 11 points in each set and 120 seconds between sets. The player is free to drink fluids during these time periods, to which must be added the possibility of asking the referee for permission to take in fluids in the break between points. According to Chen and Chen [33] the mean length of time that a badminton point lasts is 8.4±0.2 s with a break between points of 16.5±0.5 s, so the ratio between play and rest is 1:2.

Hamouti et al. [18] found higher dehydration levels than in our study in indoor soccer (1.2±0.8%) and basketball (1.1±0.5%) although volleyball players had similar dehydration values (0.4±0.6%). As mentioned before, volleyball with its play characteristics (size of court and type of effort) would be the indoor sport most similar to badminton, also with regard to access to fluids and the rest intervals during play. The players did not reach the critical value of 2% which can affect sports performance in any of the sports analyzed by Hamouti et al. [18], which may possibly be due to the access to fluids for hydration which is possible in indoor acyclic sports compared with cyclic sports, where much higher values of percentage dehydration, between 2% and 6%, have been recorded in prolonged efforts like the marathon [34].

### Jump and Hand Grip Strength

Muscle fatigue is defined as the reduction in the maximum capacity of the muscle to generate force [35]. However we did not find pre-post game differences in the force generated during the CMJ jump or during the hand grip strength test (Figures 1 and 2). The power recorded in the jump before and after the badminton match was not modified so these data suggest that the badminton match did not produce muscle fatigue in either the lower or upper limbs. These data coincide with the results obtained by Zemkova and Hamar [36] who did not find a decrease in the height of the CMJ nor squat jump (SJ) after a professional soccer match. Similarly, several authors have found that certain exercises used to produce dehydration did not cause fatigue in maximal muscle power or strength [5], [37]–[39]. Gutiérrez et al. [5] found no decreases in hand grip strength with a level of dehydration of 1.8% neither Hoffman et al. [37] found differences in jump height in spite of having induced a level of dehydration of 1.8%. Hayes and Morse [39] found no decrease in jump height in a protocol of 5 efforts designed to generate progressive dehydration by running in a hot climate (∼48°C). On the contrary, the men’s group in our study raised the height recorded in the jump with an increase of 4.5±7.3% after the match. As happened to the men in the present study, Viitasalo et al. [38] found an increase in jump height of 7.1% in subjects with a level of dehydration of 2.5%, and concluded that dehydration generated with a diuretic method did not produce a decrease in neuromuscular performance. Equally we can state that a badminton match, which is characterized by high intensity actions during brief time periods, and a match duration between 35 and 45 minutes [33] did not produce muscle fatigue in the lower limbs nor in the force generated in a hand grip test.

The men showed higher values than the women in all the CMJ parameters analyzed (height, and mean power in the push-off phase) and in the hand grip strength test (Figure 1). The men attained 27.7% more jump height than the women in the post match recording; 17.2% more power in the push-off phase of the jump and 37.1% more hand grip strength after the match. Abian-Vicén et al. [40] found similar values to the present study in 291 physically active men and 92 physically active women, where the men recorded 27.8% more height in the CMJ than the women and 20.7% more peak power in the jump. These values suggest that the specific badminton training has not modified the gender differences presented in physically active subjects who have not had specific training. It is worthy of note that power was normalized with regard to the mass of the subjects which reduced the percentage difference with respect to the height of the jumps (from 27 to 17%) but did not eliminate the gender difference.

The height of the countermovement jump was greater than that found by different authors studying physically active subjects who did not practice sports professionally [40], [41], and it is similar to that obtained by professional basketball players [42] and slightly lower than that found by Riggs and Shephard [43] in professional beach volleyball players. Given that to date no research has provided such data on badminton players, the values shown in this study related to the CMJ and hand grip strength can serve as a reference for elite male and female badminton players at the most important moment of the season for most of them, which is the National Spanish Championship.

Maximum force production generated in the hand grip test is one of the characteristics which have been studied the most in racket sports like tennis and squash [27], [28], possibly due to its importance for handling the racket. However, there are no reference data for badminton players, whose values are higher than those recorded by Alkurdi and Dweiri [44] in 20 German male students (dominant = 300.9±85.1 N, non dominant = 290.3±69.2 N) using the same methodology as in the present study, and lower than those recorded by Ducher et al. [45] in 52 tennis players (dominant = 602.7±155.7 N, non dominant = 521.1±128.5 N). These differences may possibly be due to the fact that badminton players use a racket which weighs 3 to 4 times less than a tennis racket (badminton ∼100 g; tennis ∼350 g) so that the grip strength needed by the badminton player to handle the racket will be less than that of the tennis player. The differences found between the dominant and non dominant side in both the men’s and women’s group are worthy of mention (8.0±9.5% in the pre test), as earlier studies have shown how these differences between both arms are greater in players of racket sports than in the sedentary population [46]. The differences found in the present study are similar to those presented by Mahoney and Sharp [47] who found an asymmetry between the dominant and non dominant side of 13% in hand grip strength in squash players, or the values obtained by Ducher et al. [45] who recorded a difference of 13.5% in 52 tennis players who had practiced this sport for an average of 16.2±6.1 years. Ducher et al. [45] established a significant Pearson correlation between hand grip strength and the bone mineral content of the forearm (r = 0.81) and the bone mineral density of the forearm (r = 0.67), which indicates that the badminton players, like the tennis players, may possibly have greater bone mineral development in the dominant versus the non dominant side.

### Urine Analysis

The specific gravity of urine is often used to measure the state of hydration of athletes before exercise, being a simple, low cost and non invasive method [19]–[21], [48]. It is estimated that <1.020 is the limit for considering that an athlete is correctly hydrated [19], [49], [50], although this value may be higher in athletes with a large muscle mass [48]. In a recent study Armstrong et al. [49] corrected these values by differentiating between the reference values for the first urine in the morning (U_sg_ euhydrated = 1022–1023) and 24 hours after daily activity (U_sg_ euhydrated = 1015–1017). In the present study we found that 90.9% of the players arrived with values lower than 1.020. After the competition, the specific gravity of the urine was not modified, because the players did not dehydrate (dehydration: women = 0.32±0.83%; men = 0.37±0.50%) and also because U_sg_ is a marker which involves some delay in revealing dehydration [50].

After intense exercise, urine becomes more acid and there is an increase in ammonia excretion which leads to a decrease in pH. This urine acidity occurs 10 minutes after ceasing exercise and it is maintained for up to 50–90 minutes afterwards [51]. The badminton match caused a decrease in the pH of the urine which was significant in the men’s group (pre = 7.20±1.21; post = 6.25±0.87, p<0.05) and tended to be significant in the women’s group (pre = 7.20±1.08; post = 6.28±1.05, p = 0.06). Changes in urinary pH are mainly determined by blood pH and thus urinary pH may be an indicator of how extreme the blood pH modification has been [52]. Badminton is a sport which requires multiple efforts with a short duration but high intensity [33] which favor the accumulation of lactic acid [16].

Different studies have analyzed how intense exercise influences urinary pH [23], [24], [52]. Some of these studies show different possibilities for countering or delaying the acidosis caused by exercise, like taking bicarbonate [24] or consuming an alkaline diet [52] before beginning exercise, with less than conclusive results with regard to improving performance. Rios-Enriquez et al. [52] found that no improvement was evident in performance in an anaerobic high intensity test after consuming an alkaline diet, but that there was a decrease in urinary acidity after exercise. Carr et al. [24] also found an increase in urinary pH after exercise due to taking 0.3 g/kg of bicarbonate. The improvement in performance in a 2000 m rowing test was not significant when only bicarbonate was used, but it was significant when it was combined with 6 mg/kg of caffeine. McInnis et al. [23], analyzed the influence of 4 different types of exercise on urinary pH (3× Wingate of 60 seconds, 3×400 m sprinting, cycling at 90% of the aerobic threshold and running at 90% of the aerobic threshold). These authors found no significant differences, but did however see a tendency to a more marked decrease in pH in the higher intensity efforts (Wingate and sprint). In the present study, players had to perform a great number of repeated maximum intensity efforts during the matches (∼40 minutes) which produced higher urine acidity.

The badminton match also produced different signs of imbalance in the various urinary parameters which were analyzed: leukocyturia, an increase in the presence of nitrites, proteinuria and an increase in the presence of erythrocytes and glucosuria in the men’s group (Table 1). Numerous studies state that high intensity exercise causes the appearance of hematuria and proteinuria, due to an alteration in renal function [23]. Poortmans [53] suggests that post exercise proteinuria arises as a result of the increase in glomerular permeability as, if the exercise is prolonged, red blood cells may be lost via the urine. The decreased blood flow to the kidneys is proportional to the intensity of the exercise, with a 30% reduction for exercise at 50% of VO_2_ max and a 75% reduction with exercise at 65% of VO_2_ max [54]. The drop in blood pressure causes a decrease in blood flow to the kidneys and an increase in the passing of red blood cells and proteins into the urine causing hematuria and proteinuria. The proteinuria and hematuria recorded after the badminton match are a consequence of performing high intensity exercise [23] and give an idea of the high intensity involved.

On the competition day, 60% of the players analyzed revealed values higher than 25 mg of protein per dl of urine proteins. Similar responses in urinary protein concentration have been recorded before in other types of exercise. Gur et al. [25] found that 73.3% of runners revealed proteinuria after completing a half marathon while Boileau et al. [22] found proteinuria in 30% of 383 runners after completing a marathon. McInnis et al. [23] found an increase in protein concentration after 3 series of 400 m at maximum intensity, however they did not find changes in urinary protein concentration when exercise was performed at a lower intensity than the aerobic threshold, which situates badminton matches in the category of high intensity anaerobic activities. The increase in the players’ urinary erythrocyte concentration may be due to the fact that prolonged and exhausting effort can cause an increase in the destruction of the red blood cells as a consequence of the compression of the capillaries by the muscle contractions, the increase of the speed of blood flow as well as the impacts suffered by the feet when absorbing the shocks of the constant jumps and changes of direction involved in the matches [26].

### Conclusions

The badminton players’ sweat rate was 1.02 l/h in the women and 1.14 l/h in the men, values similar to those recorded in other acyclic indoor sports played at a neutral environmental temperature. The badminton players came to the match with an adequate hydration level and maintained suitable hydration during the matches by an adequate fluid intake regime. These patterns prevented a level of dehydration which could have negatively influenced their performance. The badminton match did not produce muscle fatigue in the lower or upper limbs as jump height and hand grip strength were not modified. There was an evident asymmetry in hand grip strength in favor of the dominant side. No gender differences were found in the hydration parameters, however, the duration of the men’s matches was greater and they showed higher levels of power in their lower limbs and greater strength in their upper limbs. After the match, the urine analyses showed proteinuria, an increase in the presence of nitrites and erythrocytes and leukocyturia mainly produced by the high intensity of the game. Similar urinary anomalies have been observed in sports of longer duration like the half marathon or marathon.
